# Bone-derived PDGF-BB enhances hippocampal non-specific transcytosis through microglia-endothelial crosstalk in HFD-induced metabolic syndrome

**DOI:** 10.1186/s12974-024-03097-5

**Published:** 2024-04-29

**Authors:** Guanqiao Liu, Wen Shu, Yingqi Chen, Yong Fu, Shuai Fang, Haonan Zheng, Weike Cheng, Qingrong Lin, Yanjun Hu, Nan Jiang, Bin Yu

**Affiliations:** 1 Division of Orthopaedics and Traumatology, Department of Orthopaedics, Nanfang Hospital, Southern Medical University, Guangzhou, China; 2 Guangdong Provincial Key Laboratory of Bone and Cartilage Regenerative Medicine, Nanfang Hospital, Southern Medical University, Guangzhou, China; 3 Department of Trauma Orthopedics, Liuzhou People’s Hospital, Liuzhou, China; 4 Trauma Center, Department of Orthopaedic Trauma, The Second Affiliated Hospital of Hengyang Medical College, South China University, Hengyang, China

**Keywords:** Bone-brain axis, Platelet-derived growth factor-BB, Neuroinflammation, Transcytosis, High-fat diet

## Abstract

**Background:**

It is well known that high-fat diet (HFD)-induced metabolic syndrome plays a crucial role in cognitive decline and brain-blood barrier (BBB) breakdown. However, whether the bone-brain axis participates in this pathological process remains unknown. Here, we report that platelet-derived growth factor-BB (PDGF-BB) secretion by preosteoclasts in the bone accelerates neuroinflammation. The expression of alkaline phosphatase (ALPL), a nonspecific transcytosis marker, was upregulated during HFD challenge.

**Main body:**

Preosteoclast-specific Pdgfb transgenic mice with high PDGF-BB concentrations in the circulation recapitulated the HFD-induced neuroinflammation and transcytosis shift. Preosteoclast-specific Pdgfb knockout mice were partially rescued from hippocampal neuroinflammation and transcytosis shifts in HFD-challenged mice. HFD-induced PDGF-BB elevation aggravated microglia-associated neuroinflammation and interleukin-1β (IL-1β) secretion, which increased ALPL expression and transcytosis shift through enhancing protein 1 (SP1) translocation in endothelial cells.

**Conclusion:**

Our findings confirm the role of bone-secreted PDGF-BB in neuroinflammation and the transcytosis shift in the hippocampal region during HFD challenge and identify a novel mechanism of microglia-endothelial crosstalk in HFD-induced metabolic syndrome.

**Supplementary Information:**

The online version contains supplementary material available at 10.1186/s12974-024-03097-5.

## Background

Metabolic syndrome, a pathological condition characterized by abdominal obesity, insulin resistance, hypertension, and hyperlipidemia, has been widely investigated over the past two decades. Major advancements have been made in understanding the mechanism by which metabolic syndrome is involved in bone and brain disorders [1, 2]. Many studies have shown that metabolic stress, especially high-fat diet (HFD) challenge, exacerbates neuroinflammation and cognitive decline [3–5]. Our previous work also demonstrated that HFD promotes preosteoclast senescence and platelet-derived growth factor-BB (PDGF-BB) secretion into the circulation, which is one of the cause for microvascular impairment [6, 7]. However, how PDGF-BB secretion induced by HFD challenge impairs brain function and the mechanism of the bone-brain axis participation in metabolic stress-induced brain vascular impairment remain unknown. Thus, there is an urgent need to identify the mechanism by which elevated PDGF-BB caused by HFD-challenged induces brain microvascular impairment.

As immune cells of neuroectodermal origin, microglia mainly participate in clearing dead and surplus cells via phagocytosis [8]. Recent studies have shown that microglia play an important role in many disease processes, such as Alzheimer’s disease, multiple sclerosis, and Parkinson’s disease [9–11]. The occurrence of the above diseases is closely related to the impact of microglia on the blood-brain barrier (BBB) [12, 13]. The BBB is composed of endothelial cells, pericytes, and astrocytes at the interface between central and peripheral compartments. The BBB maintains brain homeostasis, regulates influx and efflux transport, and protects the brain from harmful components in peripheral circulation [14]. Any disruption within the brain or systemic components triggers BBB dysfunction and neuroinflammation, contributing to the pathogenesis of cerebrovascular disease [15].

Transcytosis is the vesicular trafficking of molecules between the luminal and abluminal cell membranes. Macromolecules are first endocytosed or internalized by vesicles on one side of the cell, trafficked in vesicles, and then exocytosed or released on the other side of the cell [16, 17]. The two major transcytosis at the BBB are ligand-specific receptor-mediated transcytosis and nonspecific caveolar transcytosis [18]. Recent studies have shown that nonspecific caveolar transcytosis was enhanced during aging and acute ischemic stroke [19, 20]. A recent study revealed that age-associated shifts in protein transcytosis from ligand-specific receptor-mediated transcytosis (RMT) to nonspecific caveolar RMT (non-RMT) may play an important role in brain aging [20]. The RMT is the primary pathway for macromolecule transport across the BBB and is required for the maintenance of brain homeostasis [21]. Certain genes such as transferrin receptor (Tfrc) and CD98hc (Slc3a2) are highly correlated with plasma uptake enhancement and RMT [20, 22]. A recent study showed that transcytosis shifts from RMT to non-RMT with the loss of pericyte coverage, high alkaline phosphatase expression, and diminished TFRC expression in brain microvascular endothelial cells [20]. This shift alters the composition of transcytotic plasma proteins and permits access to neurotoxic proteins that exacerbate the aged brain phenotype. Targeting ALPL activity significantly enhances TRFC expression and RMT [20], indicating that ALPL plays an important role in transcytotic shifts. Meanwhile, several studies have shown that the plasma levels of C-C motif chemokine ligand 1 (CCL-1), C-C motif chemokine ligand 2 (CCL-2), and vascular cell-adhesion molecule 1 (VCAM-1) are positively correlated with neurodegenerative disorder [23–25], indicating that circulating factors are important factors affecting brain homeostasis. Our previous work demonstrated that, as a new pro-aging factor, senescent preosteoclast-secreted PDGF-BB is elevated in the plasma and may cause pericyte loss during aging and HFD challenge [6]. However, whether PDGF-BB participates in HFD-induced neuroinflammation and how PDGF-BB-associated neuroinflammation induces a transcytosis shift and BBB leakage under HFD challenge remains to be determined.

In this study, we demonstrated the mechanism by which microglia induce a transcytotic shift in HFD-induced metabolic syndrome. Elevated PDGF-BB induced by HFD challenges causes microglia activation and IL-1β secretion. IL-1β enhances ALPL expression through SP1 translocation, increases the expression of non-RMT related protein (ALPL), and decreases the expression of ZO-1 in endothelial cells. Our study revealed the critical role of microglia-endothelial crosstalk in the transcytosis shift and elucidated the mechanism of elevated PDGF-BB levels in BBB impairment during HFD-induced metabolic syndrome.

## Materials and methods

### Mouse line

C57BL/6 conditional *Pdgfb* knockout (pdgfb^cKO^) and *Pdgfb* transgenic male mice (pdgfb^cTG^) were generated as previously described [6, 7, 26]. Briefly, *Pdgfb*^*f/f*^ mouse strain was purchased from Jackson Laboratory (Bar Harbor, ME, USA). *Pdgfb*^*f/f*^ mice were crossed with *Trap-Cre* mice to generate *Trap-Cre; Pdgfb*^*f/f*^ mice, which are referred to as “pdgfb^cKO^” in the text. Their male littermates of homozygous mice for *Pdgfb* flox allele are referred to as “WT” in the text. We conditionally deleted *pdgfb* expression in Trap^+^ preosteoclast through pdgfb^cKO^ mice.

The Pdgfb^cTG^ mouse line was generated by ligation of the mouse TRACP5 promoter with 2.8-kb full-length human PDGFB cDNA, followed by pronuclear injection in C57BL/6 fertilized eggs. We validated the genotype of the mice by polymerase chain reaction (PCR) analysis of genomic DNA isolated from mouse tails using primers described previously [6, 7, 26]. We conditionally overexpressed *pdgfb* expression in Trap^+^ preosteoclast through pdgfb^cTG^ mice. Male mice of the indicated ages were used in the experiments.

### Mouse model

Eight-week-old male C57BL/6 mice were purchased from the Laboratory Animal Center of the Southern Medical University (Guangzhou, China). At 10–12 weeks of age, the mice were fed a western HFD (42% fat) (TD 88,137, Harlan Laboratories, Madison, WI, USA) or a normal chow diet (CHD) for 3–4 months. All mice were housed under specific pathogen-free conditions at 23–25 °C in a 12-h light/dark cycle with *ad libitum* water and food access. All experimental procedures were approved by the Institutional Animal Care and Use Committee of the Southern Medical University.

### Bone marrow fluid preparation and enzyme-linked immunosorbent assay (ELISA) analysis

Mice were anesthetized with 2,2,2-tribromoethanol (20 mg/mL, T48402, Sigma) before euthanization. Tibias were separated and bone marrow cells were flushed out using a 25-gauge syringe needle with 100 µL phosphate-buffered saline (PBS) buffer. The cell suspension was collected and centrifuged at 1000 rpm for 5 min. The supernatant was collected and used to detect the PDGF-BB concentration using the Mouse/Rat PDGF-BB ELISA Kit (KE10034, Proteintech, China) according to the manufacturer’s protocol. The concentration was measured at 450 nm and 570 nm using a plate reader (SpectraMax i3x, Germany).

### Brain, bone section preparation, and immunofluorescence staining

Brain and bone sections were prepared as described previously [6, 7]. Briefly, after perfusion with PBS and formalin, the brains and bones were collected and separated sagittally. For brain preparation, the brain was fixed in 4% paraformaldehyde solution for 24 h at 4℃, transferred to 30% sucrose in PBS for another 48 h at 4℃, and embedded in OCT and cryosectioned coronally at 20 μm with a cryostat (CM1950, Leica). For bone preparation, the bone was fixed in 4% paraformaldehyde solution for 24 h at 4℃, transferred to 30% sucrose and 2% polyvinyl pyrrolidone in PBS for another 48 h at 4℃, and embedded in OCT and cryosectioned coronally at 10 μm with a microtome. All sections were stored at -80℃ before further staining.

For immunofluorescence staining, tissue sections were incubated with specific primary antibodies to mouse ALPL (1:20, AF2909, R&D), IBA1 (1:200, ab178846, Abcam), GFAP (1:500, ab7260, Abcam), SOX2 (1:100, ab92494, Abcam), ZO-1 (1:100, ab221547, Abcam), Rank (1:100, MA5-16153, Thermo Fisher), PDGF-B (1:100, ab23914, Abcam) for 24 h at 4℃ followed by corresponding fluorescence-linked secondary antibodies (Jackson ImmunoResearch Laboratories) for 1 h while avoiding light. The sections were mounted with DAPI (S2110, Solarbio). Sample images were captured using a confocal microscope (Zeiss LSM 980).

### Brain tissue preparation and ELISA

Brain tissue was prepared as previously described [6]. Briefly, mice were anesthetized with 2,2,2-Tribromoethanol (20 mg/mL, T48402, Sigma) before euthanization. The hippocampus, cortex, and thalamus were then separated under a microscope. After weighing, the tissues were placed in lysis buffer (Whole Cell Lysis Kit, KGP250, KIRGEN, China) and homogenized using a homogenizer and sonic dismembrator. After centrifuging at 12,000 rpm for 15 min, the supernatant was collected, and protein concentration was measured using Pierce™ BCA Protein Assay Kits (23,225, Thermo Fisher) according to manufacturer’s protocol. Then, 200 µg of the total proteins per sample were used to detect TNF-α, IL-1β, and IL-6 concentration using mouse TNF-α ELISA Kit (EK282, Multi Sciences, China), mouse IL-1β ELISA Kit (EK201B, Multi Sciences, China), and mouse IL-6 ELISA Kit (EK206, Multi Sciences, China) according to manufacturer’s protocol. The concentration was measured at 450 and 570 nm using a plate reader (SpectraMax i3x, Molecular Device, USA).

### Behavior test

The Morris Water Maze test was performed as described previously, with modifications [27]. Briefly, a 120-cm circular pool, platform, and camera were employed. The pool was filled with water and kept at 23 ± 1 °C. The platform was hidden 1 cm below the water surface. The camera was connected to a tracking system for recording and analysis. On the first two days, the mice underwent visible platform training of six trials per day. Animals that failed to learn during visible platform training were excluded. After two days of training, the water was opacified with milk powder. Six probe trials of the hidden platform test were performed each day for a total of 18 trials over 3 consecutive days. Swimming paths were tracked, and the average time to reach the platform was recorded using ANY-maze video tracking software (Stoelting). The platform tests were performed for five consecutive days. All behavioral tests were performed under identical environmental conditions. At least ten mice per group were included in the experiments. For acclimation, the mice were transferred to the testing room two days before the MWM test and kept in the testing room during the entire test.

Novel object recognition was performed as described previously [6]. Briefly, the mice were tested in 20 cm × 20 cm boxes. Each mouse was allowed to familiarize itself with the box for 10 min on day 1. On Day 2, each mouse was placed in a box with two identical objects (a cup or whistle) and allowed to explore for 10 min. After 10 min, the mice were discarded and returned to their cages. Thirty minutes later, one of the objects was replaced by a novel object. The mice were then placed back into the box and allowed to explore for 5 min. The time spent investigating the objects was measured using the Cleversys Topscan automatic tracking software (Clever Sys Inc., Reston, VA, USA).

### Cell culture, treatment, and conditioned medium (CM) collection

BV-2 cells (a microglial cell line) were purchased from the ATCC. BV-2 cells were cultured in DMEM and supplemented with 10% FBS and 1% penicillin/streptomycin. BV-2 cells were treated with different concentrations of PDGF-BB (20 and 50 ng/mL) for 3 days. The culture medium was collected and centrifuged at 6000 rpm for 5 min. Then, the supernatant was collected and filtered by Millipak® 0.22 μm filter. Next, the CM was stored at -80℃ for further study.

The human umbilical vein endothelial cell lines (HUVECs) was purchased from ATCC and cultured in 6 wells plate with DMEM, 2% FBS, and 1% penicillin/streptomycin. HUVECs were treated with IL-1β (10 ng/mL, 10,139-HNAE, Sino Biological) or with 1 mL of CM + 1 mL of growth medium for 1 d and harvested for further analysis.

Human brain microvascular endothelial cells (HBMECs) were obtained from Dr. Tianshi Que and purchased from Cell Systems (Seattle, WA, USA). They were cultured in 6-well plates in complete classical medium (Cell Systems) supplemented with 10% serum and culture boost (Cell Systems). HBMECs were treated with IL-1β (10 ng/mL, 10,139-HNAE, Sino Biological) or combined with ALPL inhibitor (SBI-425, 50 µM, HY-124,756, MCE) for 1 d and harvested for further analysis.

### CM-ELISA

Hundred microliters of ′CM per well was used to detect IL-1β concentration using human IL-1β ELISA kit (70-EK201BHS, Multi Sciences, China) according to manufacturer’s protocol. The concentration was measured at 450 and 570 nm using a plate reader (SpectraMax i3x, Molecular Device, USA).

### Chromatin immunoprecipitation (ChIP)

Proteins collected from HUVEC were subjected to ChIP using a Pierce Agarose ChIP Kit (Cat. 26,156, Thermo Fisher, USA). in accordance with manufacturer’s instructions. Immunoprecipitation was performed using a valid SP1 antibody (rabbit anti-human, 5931 S, CST, USA). The kit provided an RNA polymerase II antibody as a positive control. The primers used for PCR in the ALPL promoter region were shown as follows: forward, 5′-CAGGCTCGCTGAGAGAGGAAG-3′ and reverse, 5′-GGGAGCGGAGCTAGGAACG-3′; GAPDH primer was provided in the kit.

### Total protein preparation and Western blotting

The cells were washed three times with PBS and lysed in radioimmunoprecipitation assay buffer containing proteinase inhibitors. After BCA total protein quantification, 30 µg of total proteins were loaded on 10% SDS-PAGE gels and then transferred to the PVDF membrane. After being blocked with 5% milk solution at room temperature for 1 h and washed with 1 × tris-buffered saline tween three times, membranes were then incubated with the primary antibodies overnight at 4℃, and then further incubated with HRP-conjugated anti-rabbit secondary antibody (HA1001, Huabio, Hangzhou, China) for 1 h at room temperature. All the blots were visualized using ECL (Millipore Sigma, Burlington, MA, United States) and detected using BLT (GelView 6000 Pro, Antpedia, Guangzhou, China). The following primary antibodies were used: ALPL (1:1000, 11187-1-AP, Proteintech, China), ZO-1 (1:1000, 21773-1-AP, Proteintech), t-Claudin5 (1:500, 29767-1-AP, Proteintech), p-Claudin5 (1:1000, PA5-105058, Thermo Fisher, USA), Occludin (1:1000, 27260-1-AP, Proteintech), GAPDH (1:5000, ET1601-4, Huabio), and β-tubulin (1:5000, ET1602-4, Huabio).

### RNA purification, quantitative real-time PCR, and gene array analysis

Total RNA for qRT-PCR was purified using TRIzol (Invitrogen, 15,596,026) and the RNeasy Mini Kit (QIAGEN, 74,014) according to the manufacturer’s protocol. Complementary DNA (cDNA) was prepared with random primers using the SuperScript First-Strand Synthesis System (Invitrogen) and analyzed with Hieff^®^ qPCR SYBR Green Master Mix (11202ES08, YEASEN) in the thermal cycler with two sets of primers specific for each targeted gene. Target-gene expression was normalized to glyceraldehyde-3-phosphate dehydrogenase (GAPDH) messenger RNA, and relative gene expression was assessed using the 2^−ΔΔCT^ method. The primers used for qRT-PCR were as follows:

For BV-2 cell line:

- TNF-α: Forward: 5′-CAGGCGGTGCCTATGTCTC-3′,
- Reverse: 5′-CGATCACCCCGAAGTTCAGTAG-3′.
- IL-1β: Forward: 5′-GAAATGCCACCTTTTGACAGTG-3′,
- Reverse: 5′-TGGATGCTCTCATCAGGACAG-3′.
- IL-6: Forward: 5′-TAGTCCTTCCTACCCCAATTTCC-3′,
- Reverse: 5′-TTGGTCCTTAGCCACTCCTTC-3′.
- GAPDH: Forward: 5′-AGGTCGGTGTGAACGGATTTG-3′,
- Reverse: 5′-GGGGTCGTTGATGGCAACA-3′.
- For HUVEC:
- ALPL: Forward: 5′-ACTGGTACTCAGACAACGAGAT-3′,
- Reverse: 5′-ACGTCAATGTCCCTGATGTTATG-3′.
- GAPDH: Forward: 5′-GGAGCGAGATCCCTCCAAAAT-3′,
- Reverse: 5′-GGCTGTTGTCATACTTCTCATGG-3′.

### Microglia morphology, density analysis, and percentage area analysis

Microglial morphology analysis was performed as previously described [28]. Briefly, morphological analyses of microglia were performed using ImageJ software (National Institutes of Health, USA). The hippocampal area was selected, and the perimeter and ferret diameter of the microglia were calculated to analyze their morphology.

Microglial density analysis was performed using the ImageJ software. The stained image was loaded into the software, and microglial counts in the hippocampal area and hippocampal volume were calculated. Microglial density was expressed as the number of microglia in the hippocampal area per mm^3^ of the hippocampal volume.

Percentage area analysis was performed using ImageJ software. The staining image was loaded in the software, and the area of positive cells was selected through “threshold” and analyzed through “Analysed.” Data were calculated as positive area per the total area of the image.

### Image acquisition

Three images were acquired per tissue and three tissues were stained per mouse. The 10× and 20× magnification were used to capture the images. Numbers were used instead of group names to label sections and cell samples. Each image was analyzed three times, and the average was calculated to avoid bias.

### Statistics

Data were analyzed using GraphPad Prism 8 (GraphPad Software Inc., La Jolla, CA, USA). Firstly, data were tested for normality via “Analyze” function with “Normality and Lognormality Test” section. For normally distributed data, the differences between the two groups were tested for statistical significance using an independent sample two-tailed *t*-test. For data that were normally distributed and when there were more than two groups, one-way analysis of variance (ANOVA) was used with Tukey’s post-hoc comparison test. Where data were not normally distributed, Mann–Whitney U tests were used to determine statistical significance. In the in vivo studies, five mice were used per group (*n* = 5), and each dot represents an individual mouse. In the in vitro studies, experiments were repeated independently at least three times using the BV-2 cell line, HUVEC, and HBMEC. A dot in the control group represents an independent replicate experiment, and a dot in the experimental group represents a fold-change compared to the corresponding control group. For the scatter plot, Pearson’s correlation test was used to test the statistical significance. Values of *p < 0.05, **p < 0.01, and ***p < 0.001 were considered statistically significant.

## Results

### Preosteoclast-secreted PDGF-BB elevation is correlated with cognitive impairment, neuroinflammation and transcytosis shift in mice hippocampus during HFD challenge

**Fig. 1:**
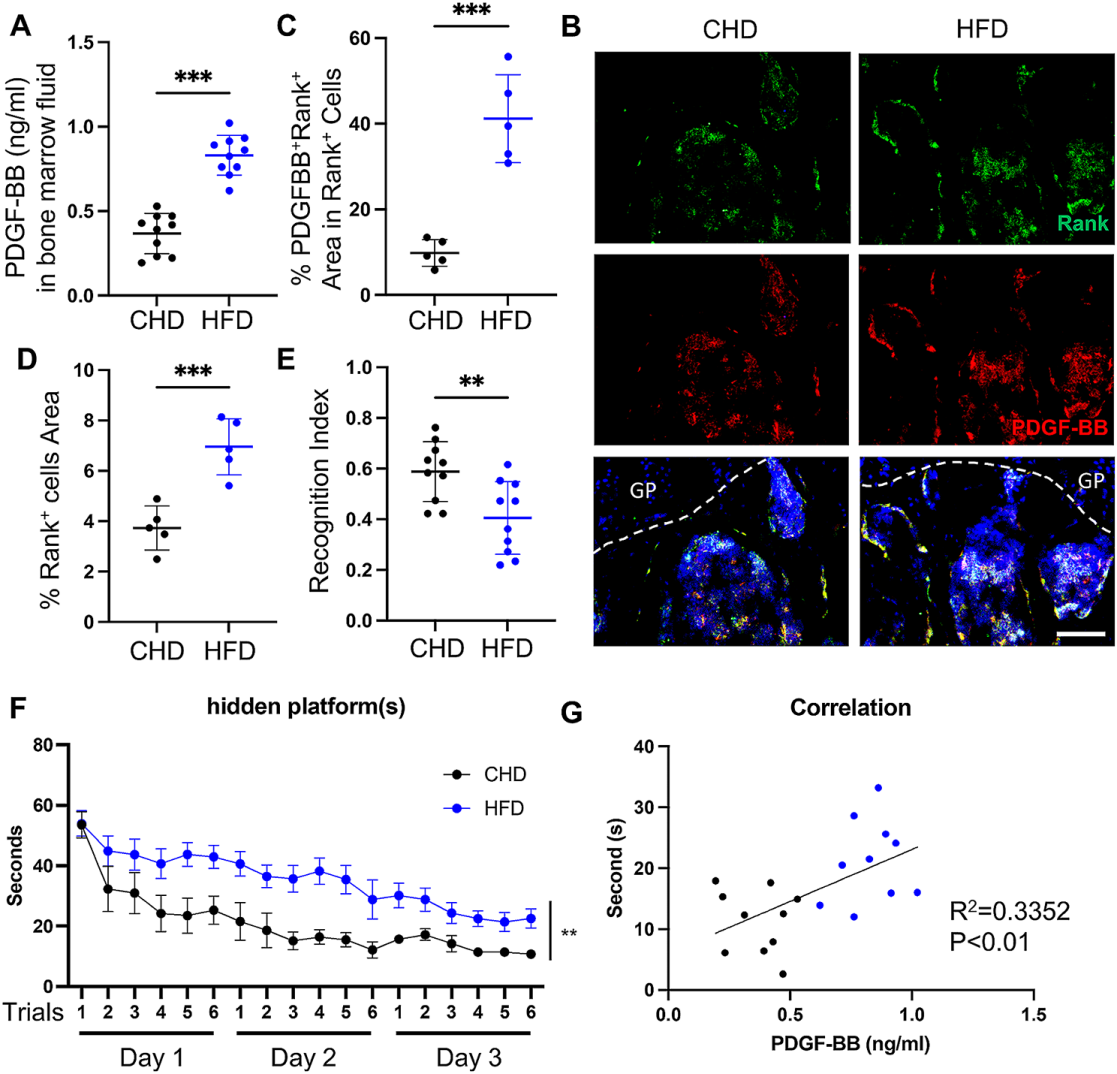
Preosteoclast-secreted PDGF-BB levels are associated with cognitive impairment in HFD-challenged mice. 3 months C57BL/6 WT mice fed CHD or HFD for 4 months. (**A**) ELISA analysis of bone marrow fluid PDGF-BB concentrations in CHD and HFD mice. (**B**) Representative confocal images of Rank (green) and PDGF-BB (red) double-immunofluorescence staining in the bone marrow of CHD and HFD mice. DAPI stains nuclei as blue. Scale bar, 50 μm. (**C-D**) Quantification of the percentage of Rank^+^ preosteoclast area and the percentage of Rank^+^ PDGF-BB^+^ area in Rank^+^ preosteoclast in the bone marrow. *n* = 5. (**E-F**) Mice were tested for novel object recognition (**E**) and Morris water maze (**F**). *n* = 10. Data are shown as the mean ± SEM. (**G**) Spearman correlation of time consumption in hidden platforms and PDGF-BB concentration in bone marrow in CHD and HFD mice. Data are shown as the mean ± SD, *p < 0.05, **p < 0.01, ***p < 0.001, as determined by unpaired two-tailed Student’s t-test

Previously studies have found that preosteoclast are a major source of PDGF-BB secretion in the bone marrow [26, 29]. Our previous study found that elevated circulating PDGF-BB levels are a key factor in cognitive impairment during aging and HFD challenge [6]. However, whether HFD-induced PDGF-BB elevation is directly caused by PDGF-BB secretion from preosteoclast is still unknown. Our results showed that the concentration of PDGF-BB in the bone marrow fluid of HFD mice was significantly higher than that in the bone marrow fluid of CHD-fed mice (Fig. 1A). The percentage area of Rank^+^ preosteoclast was elevated nearly two-folds (Fig. 1B-C) in the bone marrow of the HFD group, whereas the percentage area of PDGF-BB in Rank^+^ preosteoclast elevated nearly four-folds in the bone marrow of the HFD group (Fig. 1B, D). These results indicated that the elevated PDGF-BB concentration in the circulation was mainly caused by the increased secretion of preosteoclast. Many studies have revealed that HFD causes cognitive impairment in mice [5, 30]. Consistently, we found that HFD mice showed a lower recognition index in novel objective recognition (Fig. 1E) and required a longer time to reach the hidden platforms in the Morris water maze (Fig. 1F). Meanwhile, the time consumption of the hidden platforms was strongly correlated with the PDGF-BB concentration in the bone marrow (Fig. 1G). These results indicated that the elevated PDGF-BB concentration in the HFD challenge was mainly secreted by preosteoclast and correlated with cognitive impairment.

**Fig. 2:**
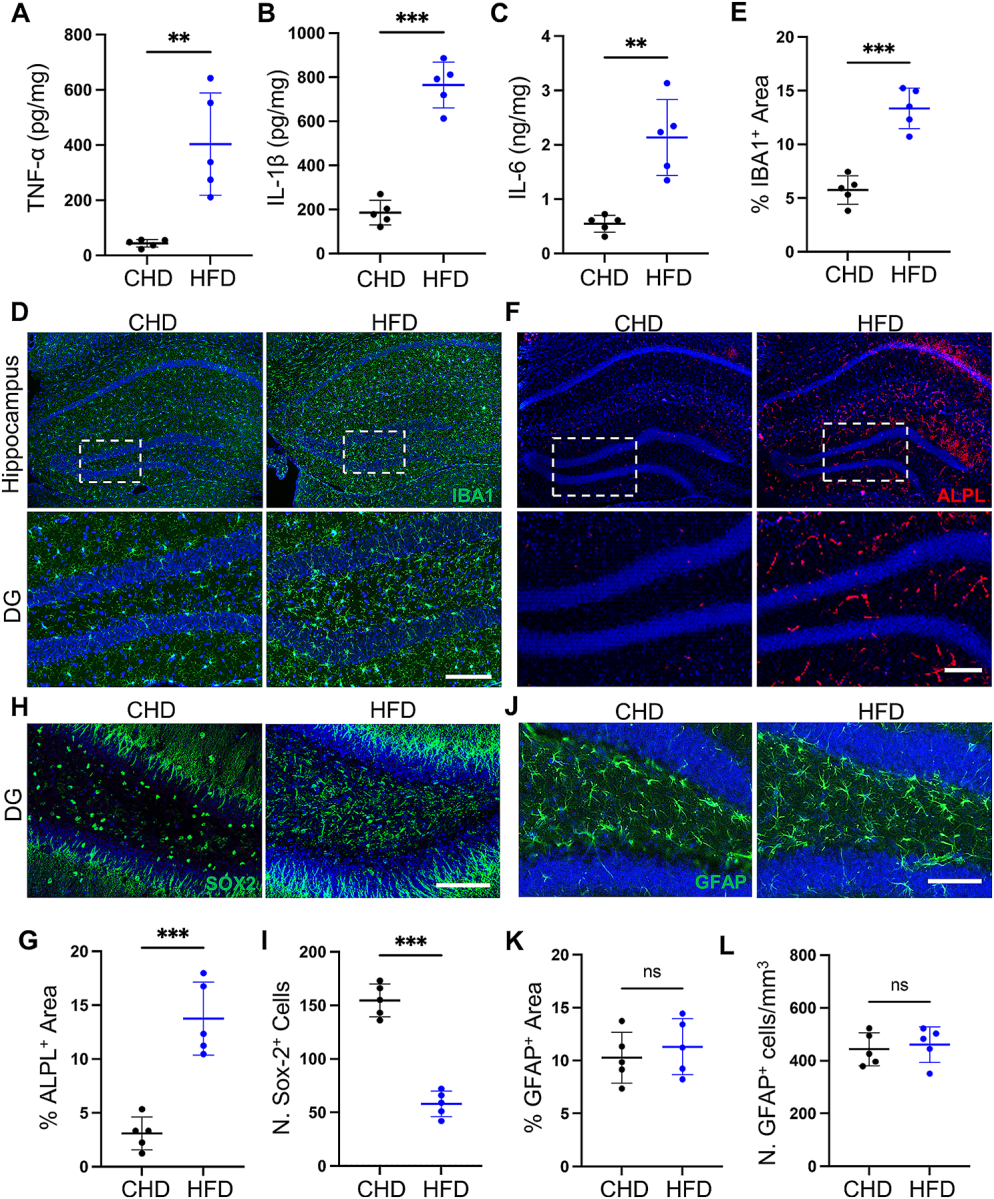
Significant increase in inflammation and non-RMT shift in the hippocampus during HFD challenge. (**A-C**) ELISA analysis of hippocampus lysate TNF-α (**A**), IL-1β (**B**) and IL-6 (**C**) concentrations in CHD and HFD mice. (**D, F, H, J**) Representative immunofluorescence images of DG region of hippocampus from CHD and HFD mice using antibodies against IBA1 (**D**), ALPL (**F**), SOX2 (**H**) and GFAP (**J**). DAPI stains nuclei as blue. Scale bar, 100 μm. (**E, G, I, K**) Quantification of IBA1^+^(**E**), ALPL^+^(**G**), SOX2^+^(**I**) and GFAP^+^(**K**) signal-covered area in the hippocampus using Image J. *n* = 5. (**L**) Quantification of the number of IBA1^+^ cells in the hippocampus region using Image J. *n* = 5. Data are shown as the mean ± SD, **p < 0.01, ***p < 0.001, as determined by unpaired two-tailed Student’s t-test

Neuroinflammation and transcytosis shifts from ligand-specific RMT to non-RMT caveolar transcytosis are two phenotypes that occur during brain aging [20, 31]. However, whether these two phenotypes participate in HFD- and PDGF-BB-induced cognitive impairments requires further investigation. To investigate whether HFD challenge causes hippocampal neuroinflammation, we separated the hippocampal tissue from the mouse brains and detected the concentration of inflammatory factors. The results showed that TNF-α, IL-1β and IL-6 concentrations are highly increased in HFD-challenged mice (Fig. 2A-C). We also detected high-density and significant microglial activation in HFD-challenged mice compared with that in the CHD mice (Fig. 2D-E, Supplemental Fig. 1A-C).

Transcytosis shift occurs alongside a loss of the RMT-specific receptor TFRC and increases in caveolin-1 and ALPL, which are molecular targets that enhance non-RMT [20]. We also detected upregulated expression of the non-RMT marker ALPL in the hippocampal region of HFD-challenged mice (Fig. 2F-G). Meanwhile, the number of SOX2^+^ neural progenitor cells was also significantly decreased in HFD-challenged mice (Fig. 2H-I), consistent with greater cognitive impairment in HFD-challenged mice. This indicates that microglia may be key cells in neuroinflammation and transcytosis shifts in HFD-induced metabolic syndromes. As another type of immune cells, astrocytes also play a crucial role in maintaining the homeostasis of extracellular fluids and exhibit dynamic activities crucial for neural circuit function, neurological function, and behavior [32]. However, we found that the number and area of astrocytes did not change during HFD challenge (Fig. 2J-L). These results indicate that HFD-induced PDGF-BB secretion from preosteoclast is correlated with cognitive impairment, inflammation, and transcytosis shifts in the mouse hippocampus and that these changes most likely start with microglia.

### HFD-induced elevation of PDGF-BB in the hippocampus plays a major role in inflammation in the brain

**Fig. 3:**
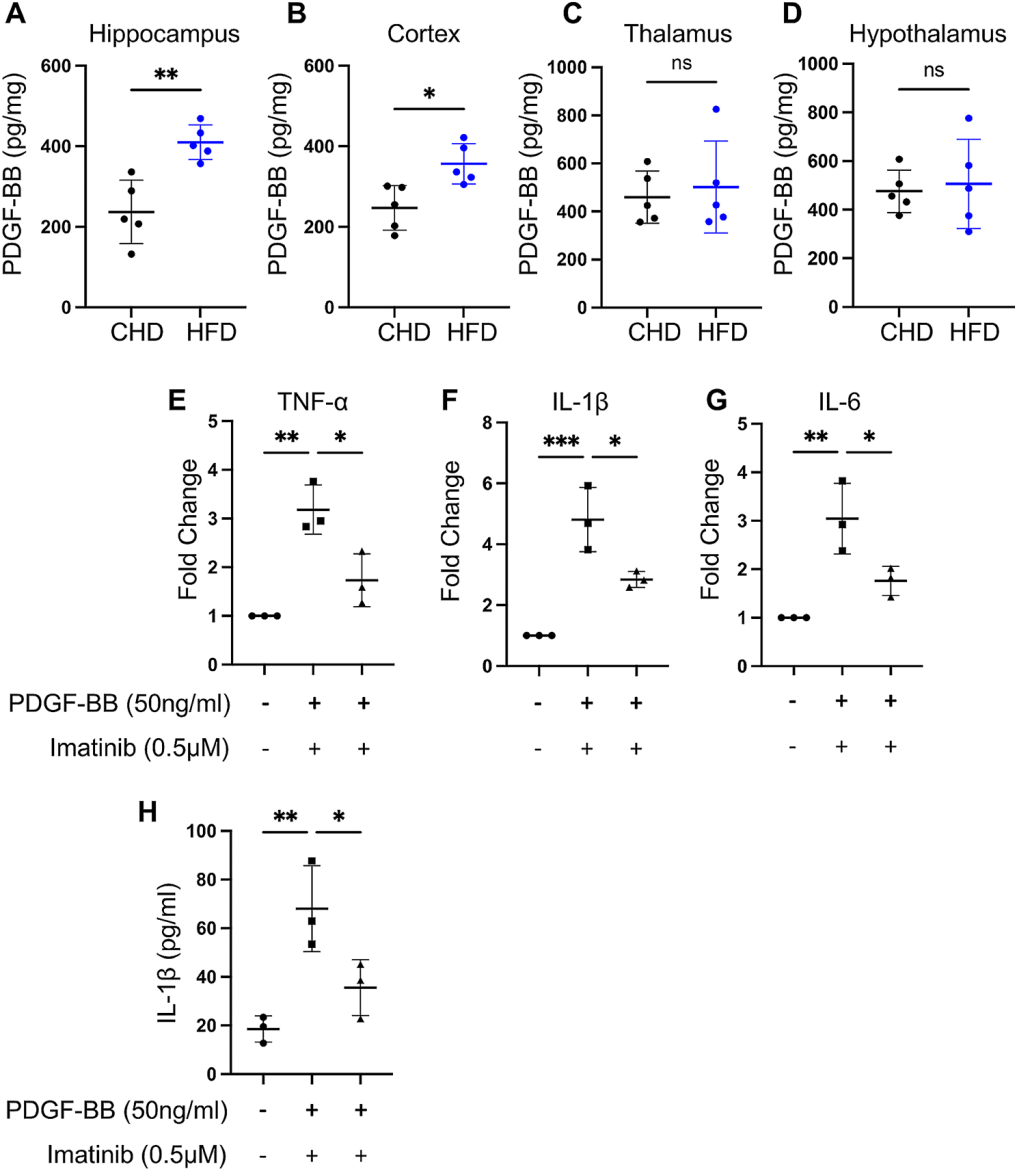
PDGF-BB elevation in the hippocampus enhances the secretion of inflammatory cytokines in macroglia through the PDGFRβ signaling pathway. (**A-D**) ELISA analysis of PDGF-BB concentration in the hippocampus (**A**), cortex (**B**), thalamus (**C**) and hypothalamus (**D**) lysate in CHD and HFD mice. *n* = 5. (**E-H**) BV-2 cell lines were treated with rh-PDGF-BB (50ng/ml) or combined with imatinib (0.5µM) for 24 h. Quantitative real-time PCR analysis of TNF-α (**E**), IL-1β (**F**) and IL-6 (**G**) mRNA expression. *n* = 3. (**H**) ELISA analysis of IL-1β concentration in conditioned medium. Data are shown as the mean ± SD, **p < 0.01, ***p < 0.001 as determined by unpaired two-tailed Student’s t-test (for two-group comparison) or One-way ANOVA (for multiple-group comparison)

Several clinical studies have shown that PDGF-BB elevation is correlated with Alzheimer’s disease, Parkinson’s disease, and dementia [33, 34]. Similarly, our previous studies showed that elevated circulating PDGF-BB is a major factor causing aortic stiffness and brain microvascular impairment during aging [6, 7]. Meanwhile, we found significant PDGF-BB elevations in the plasma and serum and restored PDGF-BB concentration by deleting Pdgfb from preosteoclasts during HFD challenge [6]. To further investigate whether PDGF-BB participates in HFD-induced neuroinflammation, we measured PDGF-BB concentrations in the hippocampus, cortex, thalamus, and hypothalamus of HFD and CHD mice. Our results showed that, compared with the same subarea in the CHD group, the PDGF-BB concentration in the HFD group was increased in the hippocampus (Fig. 3A), slightly increased in the cortex (Fig. 3B), and no change in the thalamus and hypothalamus (Fig. 3C-D). Considering that the hippocampus is associated with cognitive function, our results in Fig. 1 also showed that HFD can cause hippocampal inflammation and cognitive decline in mice. These results suggest that elevated hippocampal PDGF-BB levels are closely associated with hippocampal inflammation and cognitive impairment.

To investigate whether PDGF-BB directly induces microglia activation through the PDGFRβ signaling pathway, we treated the BV-2 cell line (mouse microglia cell line) with PDGF-BB or combined with imatinib, a PDGFRβ inhibitor. The results showed that PDGF-BB treatment significantly increased the mRNA expression of TNF-α, IL-1β and IL-6, which can be rescued by imatinib (Fig. 3E-G). ELISA results also confirm that IL-1β concentration was significantly elevated in PDGF-BB-treated CM and decreased in PDGF-BB + imatinib CM (Fig. 3H). Taken together, these results suggest that PDGF-BB elevation induced by HFD challenge may cause microglia activation and inflammatory cytokines secretion through the PDGFRβ signaling pathway.

### Manipulating PDGF-BB produced by preosteoclasts is sufficient to modulate neuroinflammation and transcytosis shifts

**Fig. 4:**
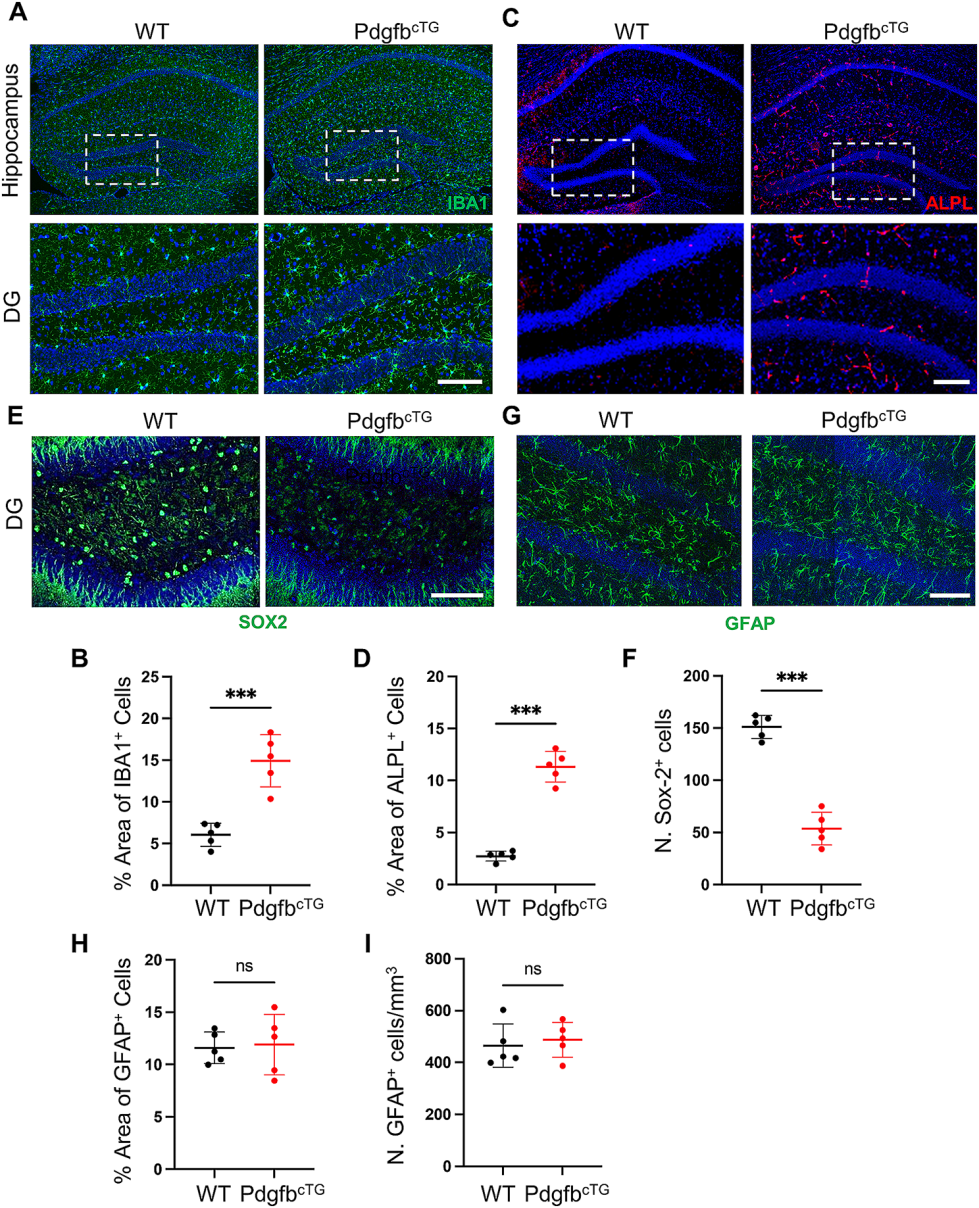
Elevated PDGF-BB produced by preosteoclasts is sufficient to induce neuroinflammation and transcytosis shift. (**A, C, E, G**) Representative immunofluorescence images of DG region of the hippocampus using antibodies against IBA1 (**D**), ALPL (**F**), SOX2 (**H**) and GFAP (**J**) in 6-month-old Pdgfb^cTG^ mice and WT littermates. DAPI stains nuclei as blue. Scale bar, 100 μm. (**B, D, F, H**) Quantification of IBA1^+^(**B**), ALPL^+^(**D**), SOX2^+^(**F**) and GFAP^+^(**H**) signal-covered area using Image J. *n* = 5. (**I**) Quantification of the number of IBA1^+^ cells in the hippocampus region using Image J. *n* = 5. Data are shown as the mean ± SD, ***p < 0.001, as determined by unpaired two-tailed Student’s t-test

To investigate whether there is neuroinflammation and transcytosis shifts in conditional Pdgfb transgenic mice (Pdgfb^cTG^), in which PDGF-BB is overexpressed in TRAP^+^ preosteoclasts and results in the elevated level of PDGF-BB [6, 7], we used IBA1 antibody to detect microglia activation and we found high density and significant microglia activation in Pdgfb^cTG^ mice compared with WT littermates (Fig. 4A-B, Supplemental Fig. 1D-F), and we also detected upregulated expression of non-RMT marker ALPL in the hippocampal region of Pdgfb^cTG^ mice (Fig. 4C-D). Our previous study showed that 6-month Pdgfb^cTG^ mice exhibit cognitive impairment [6]. As expected, the number of SOX2^+^ neurons significantly decreased (Fig. 4E-F), indicating that the cognitive impairment caused by manipulating PDGF-BB secretion may be associated with neural progenitor cell loss. However, the number and area of astrocytes did not change (Fig. 4G-I) in Pdgfb^cTG^ mice compared to their WT littermates. These results suggest that increased PDGF-BB derived from preosteoclasts can induce neuroinflammation and transcytosis shifts, consistent with the HFD-challenged phenotype.

**Fig. 5:**
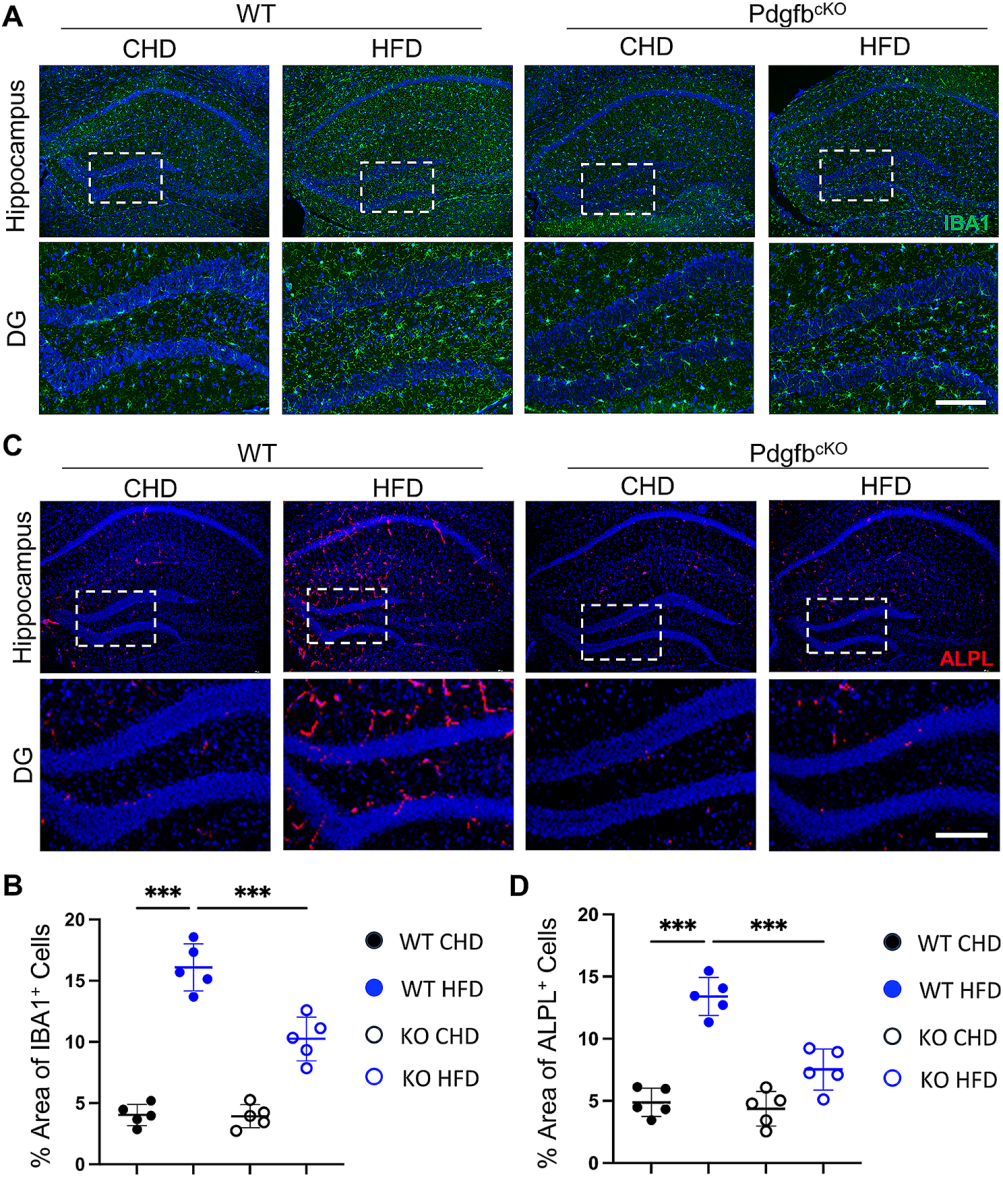
Normalizing the PDGF-BB by deleting *Pdgfb* from preosteoclasts ameliorates HFD-induced neuroinflammation and transcytosis shift. Pdgfb^cKO^ mice and WT littermates were fed HFD or CHD for 4 months, starting from 3 months of age. (**A, C**) Representative immunofluorescence images of the DG region of the hippocampus using antibodies against IBA1 (**A**) and ALPL (**C**). DAPI stains nuclei as blue. Scale bar, 100 μm. (**B, D**) Quantification of IBA1^+^(**B**) and ALPL^+^**(D)** signal covered area using Image J. *n* = 5. Data are shown as the mean ± SD, ***p < 0.001, as determined by One-way ANOVA

Our previous study showed that normalizing the level of circulating PDGF-BB using a genetic approach could restore deficits in the cerebral vasculature and cognition in HFD-challenged mice [6]. We took advantage of established conditional Pdgfb-knockout mice (Pdgfb^cKO^) [6, 7, 29], in which circulating PDGF-BB levels could be reduced by approximately 1/3^rd^. Our results showed that microglial density, activation, and ALPL expression in the hippocampus were increased in HFD-challenged mice (vs. CHD mice). However, these abnormalities were mitigated in Pdgfb^cKO^ mice under HFD challenge (Fig. 5, Supplemental Fig. 1G-I). These results indicate that manipulating the PDGF-BB produced by preosteoclasts is sufficient to modulate neuroinflammation and transcytosis shifts.

### PDGF-BB induced IL-1β secretion and non-RMT disrupts endothelial tight junction through microglia-endothelial crosstalk

**Fig. 6:**
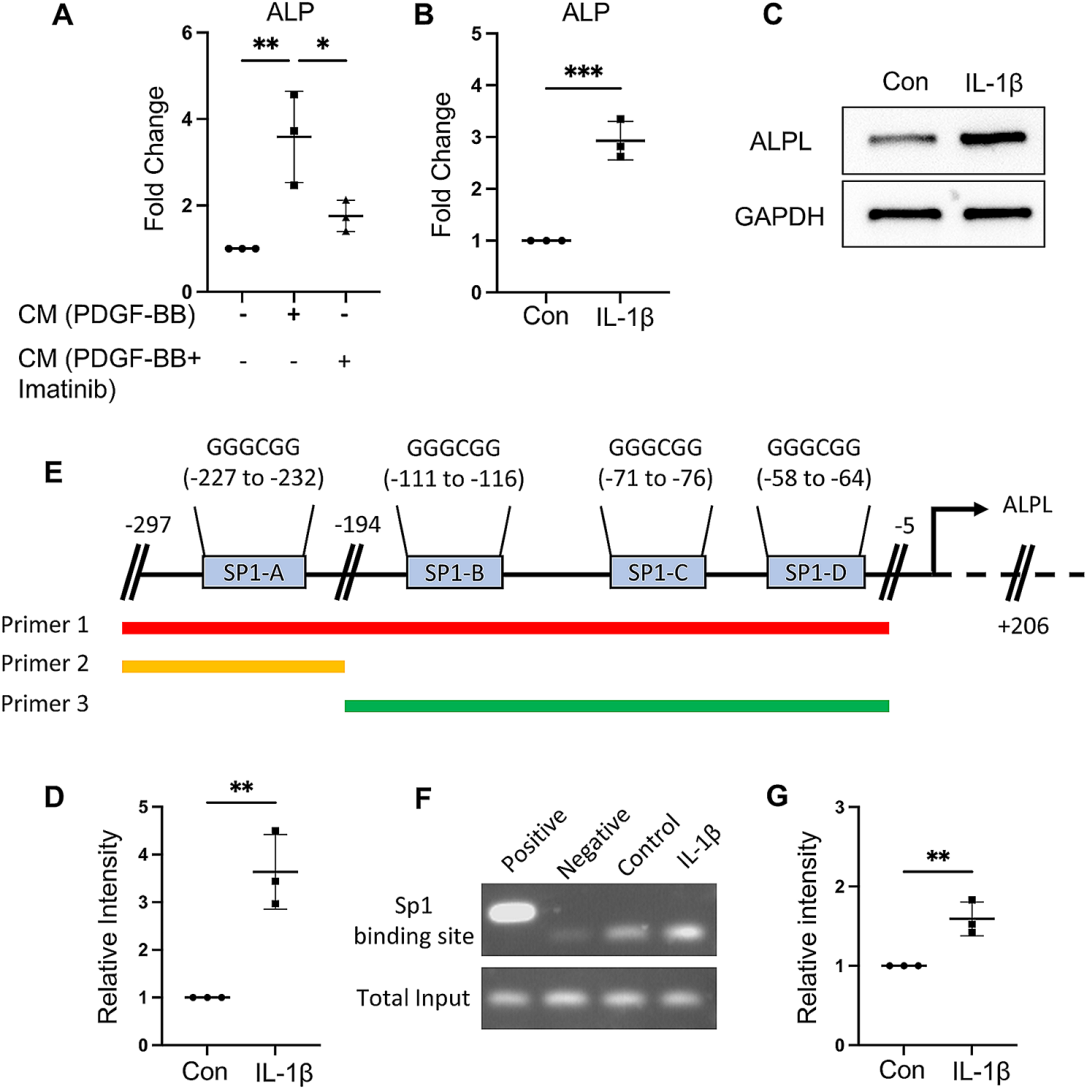
IL-1β secreted by microglia promotes ALPL transcription through SP1 translocation. (**A**) HUVEC were treated with or without BV-2 CM and BV-2 + imatinib CM for 24 h. Quantitative real-time PCR analysis of ALPL mRNA expression. (**B-D**) HUVEC were treated with or without IL-1β (10ng/ml) for 24 h. (**B**) Quantitative real-time PCR analysis of ALPL mRNA expression. (**C**) Western blot analysis of ALPL protein expression and (**D**) quantification of the relative intensity of ALPL using Image J. (**E**) Transcriptional factor prediction and primers design. (**F**) Gel image of chromatin immunoprecipitation using SP1 antibody, designed primer in PCR production of different groups. (**G**) Quantification of the relative intensity of ALPL using Image J. *n* = 3. Data are shown as the mean ± SD, *p < 0.05, **p < 0.01, ***p < 0.001 as determined by unpaired two-tailed Student’s t-test (for two-group comparison) or One-way ANOVA (for multiple-group comparison)

To determine the mechanism underlying the PDGF-BB-induced non-RMT shift, we treated BV-2 cells with PDGF-BB alone or in combination with imatinib for 24 h. Then, we collected the CM and treated HUVEC with the CM for 24 h. We found that the mRNA expression of ALPL was increased with PDGF-BB CM treatment, but slightly increased with PDGF-BB + imatinib CM treatment (Fig. 6A). Considering that large inflammatory cytokines can be synthesized during PDGF-BB treatment in BV-2 cells and that there is a relationship between ALPL and IL-1β [35], we explore if IL-1β plays a role in ALPL expression. Intriguingly, mRNA and protein levels of ALPL expression in HUVEC were significantly increased compared with the control group after being treated with IL-1β (Fig. 6B-D). These results indicated that IL-1β could enhance the expression of ALPL.

To explore how IL-1β regulates ALPL expression, we analyzed the nucleic acid sequence of the ALPL promoter region (data not shown). We identified four predicted binding sites for the transcription factor SP1 in the ALPL promoter region. We designed three pairs of primers to detect whether SP1 interacts with the ALPL promoter region (Fig. 6E). As predicted, IL-1β treatment enhanced the interaction between transcription factor SP1 and ALPL promoter region (Fig. 6F-G). These results indicated that elevated PDGF-BB induced by HFD challenges may cause microglia activation and IL-1β secretion, whereas IL-1β enhanced ALPL expression through SP1 translocation and non-RMT shift.

**Fig. 7:**
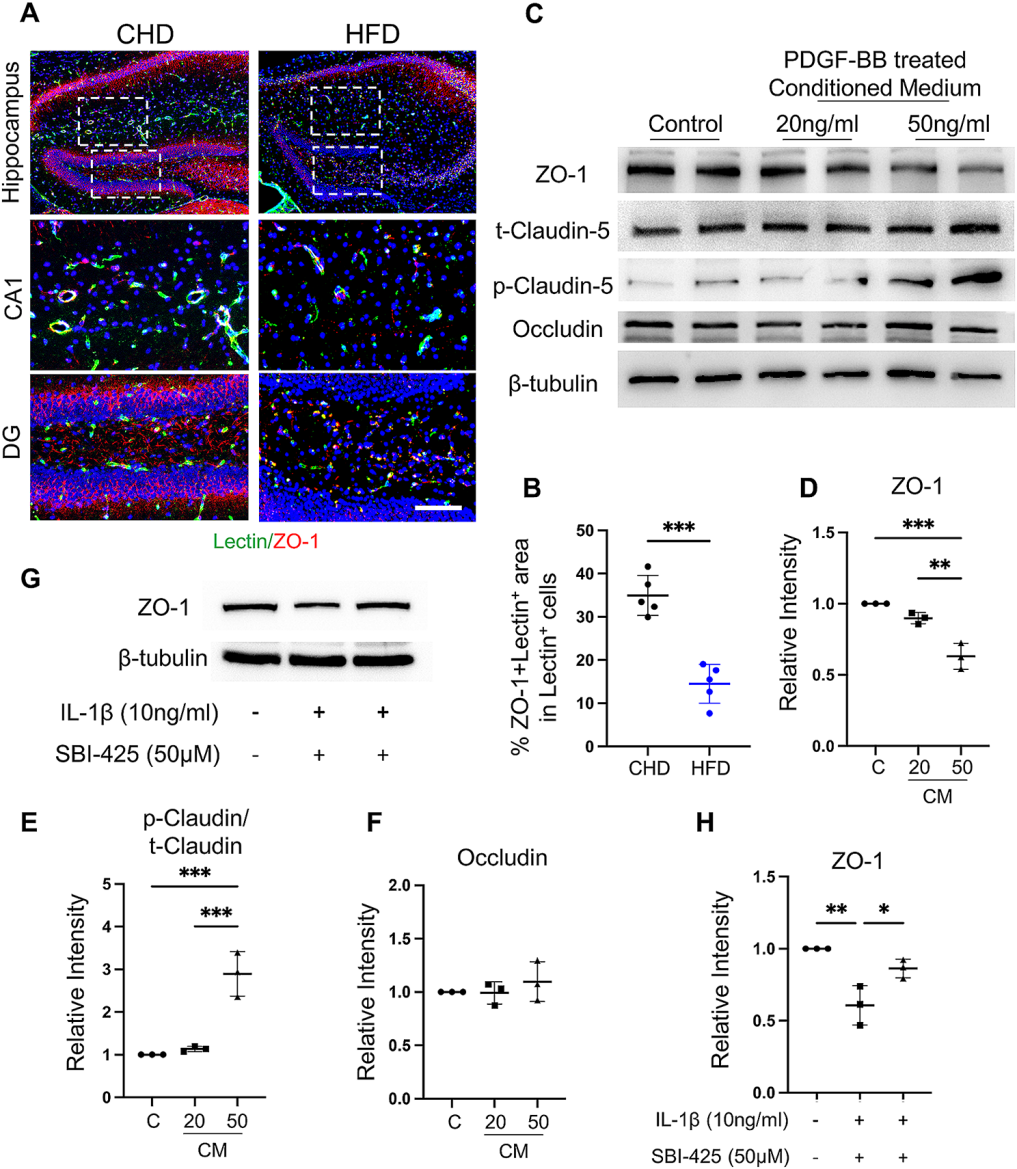
PDGF-BB-induced ALPL disrupts endothelial tight junction. (**A**) Representative confocal images of Lectin (green) and ZO-1 (red) double-immunofluorescence staining in the hippocampus area of CHD and HFD mice. DAPI stains nuclei as blue. Scale bar, 100 μm. (**B**) Quantification of percentage double positive area in Lectin^+^ microvascular in whole hippocampus area. (**C-F**) HUVEC were treated with or without BV-2 CM with 20 and 50ng/ml PDGF-BB concentrations for 24 h. (**C**) Western blot analysis of ZO-1, p-claudin-5, t-claudin-5, occludin and β-tubulin protein expression. Quantification of the relative intensity of ZO-1 (**D**), occludin (**F**) and the ratio of p-claudin-5/t-claudin-5 (**E**) using Image J. *n* = 3. (**G-H**) HBMECs were treated with IL-1β or combined with ALPL inhibitor (SBI-425, 50µM) for 24 h. (**G**) Western blot analysis of ZO-1 and β-tubulin protein expression. (**H**) Quantification of the relative intensity of ZO-1 using Image J. *n* = 3. Data are shown as the mean ± SD, **p < 0.01, ***p < 0.001 as determined by unpaired two-tailed Student’s t-test (for two-group comparison) or One-way ANOVA (for multiple-group comparison)

Many studies have shown that HFD challenges impair the BBB [36, 37]. Our previous study also showed that HFD may induce BBB leakage through persistent PDGF-BB stimulation [6]. However, the mechanism through which HFD-induced PDGF-BB secretion impairs BBB permeability remains unclear. As the most important factors in the BBB, tight junction proteins (ZO-1, claudin-5, occludin) are important in maintaining BBB permeability [38, 39]. We found that ZO-1 expression was significantly decreased in the lectin^+^ microvasculature of the hippocampal region (Fig. 7A-B). Meanwhile, decreased ZO-1 expression and an increased p-claudin-5/t-claudin-5 ratio were detected in HUVEC after treatment with different concentrations of PDGF-BB-CM (Fig. 7C-E), whereas no change was detected in the expression of occludin (Fig. 7C and F).

Although we detected the response of endothelial cells to CM or IL-1β treatment as in HUVECs, it is still not a model of the BBB. To investigate whether inhibition of ALPL could rescue IL-1β-induced tight junction loss in BBB in vitro mode, we treated human brain microvascular endothelial cells (HBMECs) with IL-1β or combined with ALPL inhibitor. The results showed that IL-1β could significantly decrease the expression of ZO-1, whereas ALPL inhibitor could rescue the IL-1β-induced ZO-1 loss (Fig. 7G-H). These results indicated that PDGF-BB-induced ALPL disrupts the expression of endothelial tight junctions.

## Discussion

**Fig. 8:**
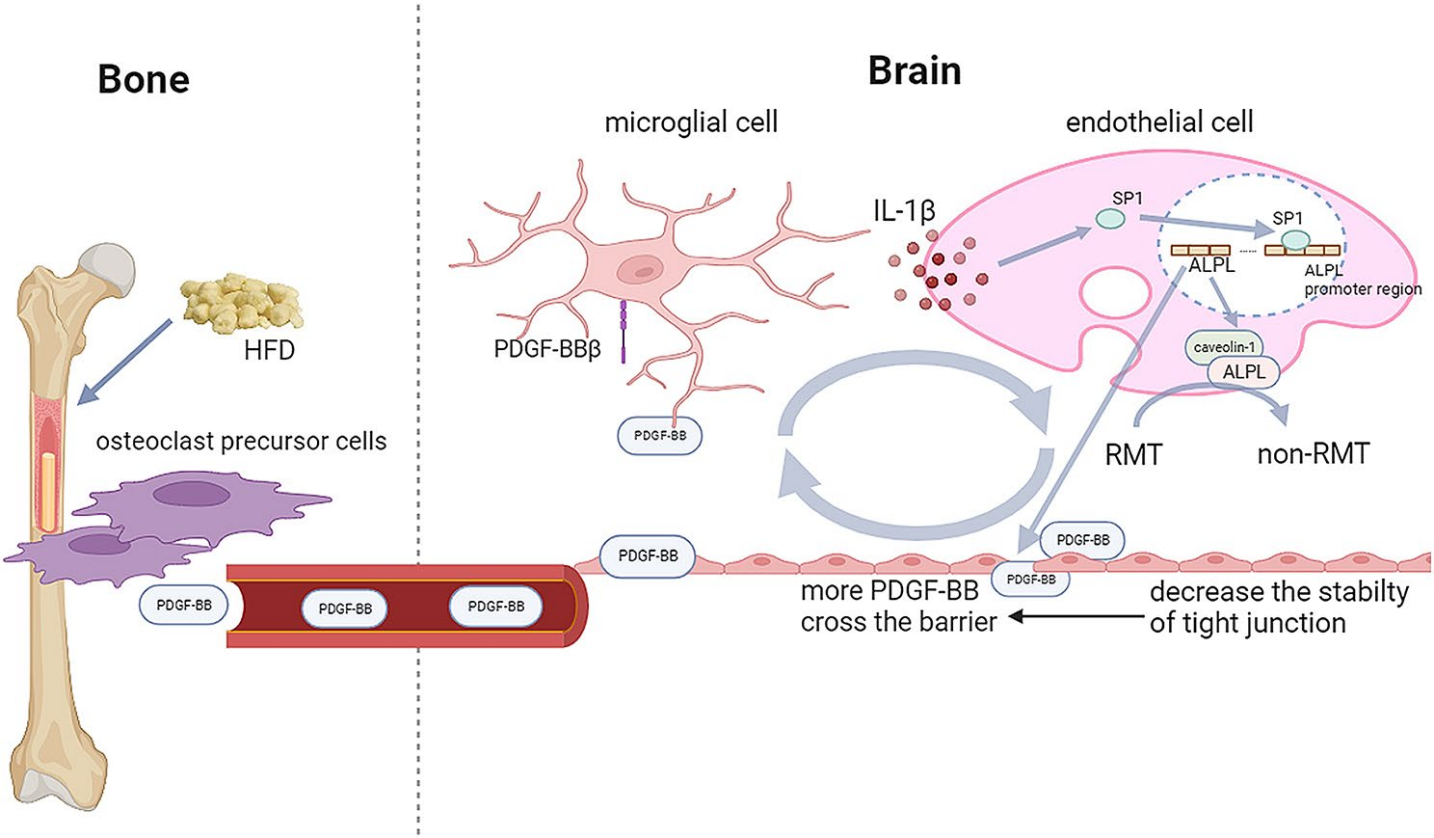
Elevated PDGF-BB induced by HFD was secreted by preosteoclast and its concentration is correlated with cognitive decline. Elevated PDGF-BB may penetrate into the brain hippocampus, causing microglia activation and IL-1β secretion. IL-1β enhanced ALPL expression through SP1 translocation, increases the expression of non-RMT related protein (ALPL) and decreases the expression of ZO-1 in endothelial cells

In this study, we identified the critical roles of PDGF-BB in cognitive decline and transcytosis shifts in HFD-induced metabolic syndrome. We showed direct evidence that elevated levels of PDGF-BB in the bone induced by HFD were secreted by preosteoclast and that its concentration was correlated with cognitive decline. Microglial activation, non-RMT shift, and neuronal loss were observed in HFD-challenged mice, whereas the number of astrocytes showed no change. This indicates that microglia may play an essential role in HFD-induced neuroinflammation. Then, we found elevated PDGF-BB levels only in the hippocampus and cortex, and inhibiting PDGFRβ signaling could alleviate the secretion of the inflammatory cytokines in microglia induced by PDGF-BB. Manipulation of PDGF-BB expression in TRAP^+^ preosteoclast is sufficient to modulate neuroinflammation and nonspecific transcytosis. Furthermore, we found that elevated PDGF-BB induced by HFD challenges may cause microglia activation and IL-1β secretion. IL-1β enhanced ALPL expression through SP1 translocation, altered RMT to non-RMT and decreased the expression of tight junction proteins in endothelial cells (Fig. 8). Therefore, we concluded that bone-derived PDGF-BB is a critical molecule that enhances non-specific hippocampal transcytosis through microglia-endothelial crosstalk in HFD-induced metabolic syndrome.

One of the most important factors in maintaining brain homeostasis is the physical BBB, which is formed by microvascular endothelial cells, pericytes, and astrocytes that restrict neurotoxic molecules in the peripheral circulation into the brain and regulate transcytosis to maintain homeostasis in the neuronal environment so that neurons can function normally. However, an increasing number of studies have demonstrated that circulatory proteins are sensitive to cognitive decline [20, 23, 40]. Many studies have shown that hyperlipidemia is one of the main risk factors that cause BBB breakdown [36, 41, 42].

Unlike the ketogenic diet, which contains low-carbohydrate and mimics starvation, the western high-fat diet contains high-carbohydrate that causes obesity and hyperlipidemia [43, 44]. In contrast to the ketogenic diet, which has neuroprotective and angiogenic effects [2, 45, 46], HFD-related hyperlipidemia and metabolic syndromes have been shown to induce neuroinflammation, BBB impairment, and cognitive decline [4, 42, 47]. Thus, great progress has also been made in alleviating HFD-induced BBB breakdown using compounds such as fibroblast growth factor 21 (FGF21), palmitoylethanolamide, and resveratrol [30, 36, 48]. Our previous study confirmed that HFD significantly increased BBB permeability and enhanced cognitive decline. Moreover, normalizing PDGF-BB levels in the circulation rescued HFD-induced cognitive impairment [6]. However, we failed to explore the mechanism by which the depletion of PDGF-BB in preosteoclast alleviates BBB breakdown and cognitive decline caused by HFD. In this study, based on our previous findings, we clarified the major neuroinflammatory cell types involved in HFD-induced preosteoclast secretion of PDGF-BB and determined the function of microglia-endothelial crosstalk in mediating the transcytosis shift and BBB permeability. Thus, normalizing PDGF-BB levels in the circulation may be an optimum strategy to attenuate BBB impairment during HFD challenge.

A recent study demonstrated an age-related shift in protein transcytosis from RMT to non-RMT in brain microvascular endothelial cells [20]. This shift alters the composition of transcytosis proteins and permits neurotoxic proteins to access the aged parenchyma to exacerbate the aged brain phenotype, with a loss of the RMT-specific receptor TFRC, as well as increases in caveolin-1 and ALPL, which are molecular targets that enhance non-RMT. Similarly, in our previous study and the current study, we observed upregulated expression of the non-RMT markers caveolin-1 and ALPL and diminished expression of the RMT receptor TFRC in hippocampal capillaries of aged, HFD-challenged, Pdgfb^cTG^ and Pdgfb^cKO^ mice ( [6] & Figs. 2F, 4C and 5C). Therefore, Pdgfb^cTG^ with elevated plasma PDGF-BB levels decreased RMT, but increased non-RMT transcytosis, mimicking the HFD-induced transcytosis shift. This new data suggests that elevated plasma PDGF-BB transmigrates across the endothelium to interact with the microglial layer, likely through increased non-RMT transcytosis [20].

Many studies have shown that HFD can induce systemic inflammation and decrease endothelial transcytosis [36, 49–52]. Similarly, we previously found that Pdgfb^cTG^ mice developed osteoarthritis [53], a skeletal disorder that results in increased levels of circulating inflammatory factors. These factors may increase the non-RMT of BBB to enable the PDGF-BB across brain endothelium and then act on its receptor PDGFRβ in microglia. It is important to verify this hypothesis in future studies. However, no studies have focused on HFD-induced transcytotic shifts. In our previous study, we showed that persistent PDGF-BB stimulation enhanced the shift from RMT to non-RMT during aging [6]. Similar to the aging phenotype, this study revealed that HFD challenge might induce a transcytosis shift by enhancing ALPL transcription, which may be a major discovery in the mechanism of HFD-induced transcytosis shift. Thus, the inhibition of RMT to non-RMT shifts using ALPL inhibitors might be an effective way to prevent cognitive decline caused by HFD-induced metabolic syndrome. In the present study, we did not intend to emphasize the role of elevated plasma PDGF-BB levels as a trigger for BBB breakdown under the HFD challenge. Instead, our data imply that elevated plasma PDGF-BB is an important systemic factor in promoting and accelerating BBB impairment and further investigates the molecular mechanism by which PDGF-BB aggravates the transcytosis shift and BBB breakdown.

It is well known that low level of PDGF-BB with low PDGF-BB/PDGFRβ signaling causes BBB impairment and cerebrovascular developmental defects [54–56]. Our previous studies demonstrated that elevated PDGF-BB also leads to cerebrovascular deficits, providing a novel component to the current understanding of the PDGF-BB/PDGFRβ signaling regulation in the BBB permeability [6]. However, the mechanisms by which PDGF-BB induces neuroinflammation and transcytosis remain unclear. This study demonstrated that PDGF-BB may directly activate the PDGFRβ signaling pathway and increase IL-1β secretion in microglia. As an inflammatory cytokine, IL-1β improved SP1 translocation and enhanced ALPL mRNA transcription, accelerating the shift from RMT to non-RMT. Similar to other studies, we also demonstrated that HFD challenge may decrease the number and stability of tight junctions, which may explain why circulating PDGF-BB can cross the brain microvascular endothelial barrier during HFD challenge.

Our previous studies have demonstrated that aberrantly elevated circulating PDGF-BB levels during aging are mainly produced by TRAP^+^ skeletal preosteoclasts [57]. Specifically, while aged mice had more than two-fold higher serum PDGF-BB levels than young mice, conditional knockout mice with *Pdgfb* deletion from preosteoclasts (Pdgfb^cKO^) had a normalized serum PDGF-BB concentration. Furthermore, increased expression of *Pdgfb* was detected in bone marrow preosteoclasts but not in peripheral blood myeloid cells in conditional *Pdgfb* transgenic mice (Pdgfb^cTG^) [57]. These results from our previous work demonstrated that elevated PDGF-BB levels in the circulation of Pdgfb^cTG^ mice are primarily produced from bone/bone marrow preosteoclasts.

However, it remains to be determined whether other types of cells in the central nervous system affect local PDGF-BB concentration. A previous study showed TRAP-like immunoreactivity in neuronal cell bodies in the rat central nervous system [58]. Similarly, we reanalyzed single-cell sequencing data from the mouse brain and found that *ACP5 (Trap)* was mainly expressed in the myelinating and myeloid lineages. In our previous studies, we examined the expression of PDGF-BB in myeloid cells in the brain tissue using our model system. Immunofluorescence staining result showed that PDGF-BB^+^ cells were detected in the bone and brain tissues of WT mice, suggesting baseline PDGF-BB expression in both tissues. However, significantly increased numbers of PDGF-BB^+^ cells were found only in the bone marrow but not in the brain of Pdgfb^cTG^ mice. In addition, the number of PDGF-BB-expressing microglial cells (TMEM119^+^ and Iba1^+^ cells) did not increase in the brain tissue of Pdgfb^cTG^ mice, indicating that local myeloid cells in the brain are not the main source of increased PDGF-BB [59]. This new finding suggests that elevated circulating PDGF-BB in transgenic mice is primarily produced by preosteoclasts in the skeletal system and is not a local effect derived from cells within the brain; manipulating PDGF-BB expression in Trap^+^ cells does not affect local PDGF-BB secretion.

It is important to understand if PDGF-BB influences astrocytic endfeet by modulating AQP4 expression. A recent study has shown that a reduction in PDGF-B signaling causes pericyte deficiency and AQP4 mislocalization in PDGFB^ret/ret^ mice [60]. Similarly, in our recent study, we found that elevated PDGF-BB in Pdgfb^cTG^ mice might activate the phosphate transporter Slc20a1 in astrocytes in the thalamic region, causing brain calcification [59]. These findings indicated that PDGF-B signaling in astrocytes is crucial for brain microvascular development. However, a previous study found that the expression of AQP4 was significantly different in the cortex and hippocampus during postnatal and adulthood [60]. Meanwhile, long-term HFD challenge could increase AQP4 density and polarization in the hypothalamus, but not in the hippocampus [61]. Considering that there was no change in the number and area of astrocytes in the hippocampal region in PDGFB^cTG^ and WT mice fed with CHD and HFD, different responses may occur in different brain subareas with respect to HFD challenge and persistent PDGF-BB elevation. A recent study focused on the brain vascular atlas showed that pericytes can be divided into two sub-groups, T-pericytes and M-pericytes, which are responsible for transmembrane transport and integrin cell surface interactions, respectively [62]. Similarly, astrocytic transcriptional identity is the most influenced by the brain region and can be divided into two clusters, where cluster 0 is mainly in the hippocampal area and cluster 1 is mainly in the cortical area [62]. As a brain region-specific astrocyte marker, TENM4^+^ astrocytes are mainly expressed in the hippocampal area rather than the cortical area [62]. These results indicate that the HFD challenge may result in different phenotypes in different brain subareas owing to the different cell subtype distributions.

Although we found that bone-derived PDGF-BB is a key molecule that enhances nonspecific hippocampal transcytosis through microglia-endothelial crosstalk in HFD-induced metabolic syndrome, several aspects require further study. First, although we verified that inhibiting PDGFRβ signaling could alleviate IL-1β secretion, the molecular involved between PDGFRβ and IL-1β remains to be explored. Second, although we found that manipulating PDGF-BB expression in preosteoclast is sufficient to modulate neuroinflammation and nonspecific transcytosis, whether the specific knockdown of ALPL in brain endothelial cells can be waived due to the impairment of HFD-induced metabolic syndrome needs to be further investigated. Third, considering the important role of ALPL in non-RMT, it is essential to overexpress ALPL in future studies in endothelial cells or transgenic mice to determine its function during HFD challenge. Lastly, although we found no significant difference in the level of circulating PDGF-BB and cognitive decline between PDGFB^cKO^ mice and their control littermates fed CHD [6], bone mass was significantly decreased, which may be due to the loss of CD31^+^EMCN^+^ H-type vessels in our previous studies [29, 57]. Therefore, it is necessary to construct inducible Cre mice for further studies.

## Conclusion

HFD-induced elevated PDGF-BB induced by HFD is secreted by preosteoclast, and its concentration correlates with cognitive decline. Elevated PDGF-BB may penetrate into the brain hippocampus, and cause microglia activation and IL-1β secretion. IL-1β enhances ALPL expression through SP1 translocation, increases the expression of non-RMT related protein (ALPL) and decreases the expression of ZO-1 in endothelial cells.

### Electronic supplementary material

Below is the link to the electronic supplementary material.

Supplementary Material 1

## Data Availability

The datasets of the present study are available from the corresponding authors upon reasonable request.

## Abbreviations

ALPL: alkaline phosphatase
BBB: brain-blood barrier
CCL-1: C-C motif chemokine ligand 1
CCL-2: C-C motif chemokine ligand 2
ChIP: chromatin immunoprecipitation
CM: conditioned medium
GAPDH: glyceraldehyde-3-phosphate dehydrogenase
HFD: high-fat diet
HBMEC: human brain microvascular endothelial cell
HUVEC: human umbilical vein endothelial cell line
IL-1β: interleukin-1β
non-RMT: nonspecific caveolar receptor-mediated transcytosis
CHD: normal chow diet
ANOVA: one-way analysis of variance
PBS: phosphate-buffered saline
PDGF-BB: platelet-derived growth factor-BB
PCR: polymerase chain reaction
RMT: receptor-mediated transcytosis
Tfrc: transferrin receptor
VCAM-1: vascular cell-adhesion molecule 1
