# Supplementary material for: Bone-derived PDGF-BB enhances hippocampal non-specific transcytosis through microglia-endothelial crosstalk in HFD-induced metabolic syndrome
Source: J Neuroinflammation. 2024 Apr 29;21:111. doi: 10.1186/s12974-024-03097-5 (PMC11057146; doi:10.1186/s12974-024-03097-5)
Supplement: Supplementary file 1 — Supplementary Material 1 [file 12974_2024_3097_MOESM1_ESM.pdf]

Supplemental Figure 1

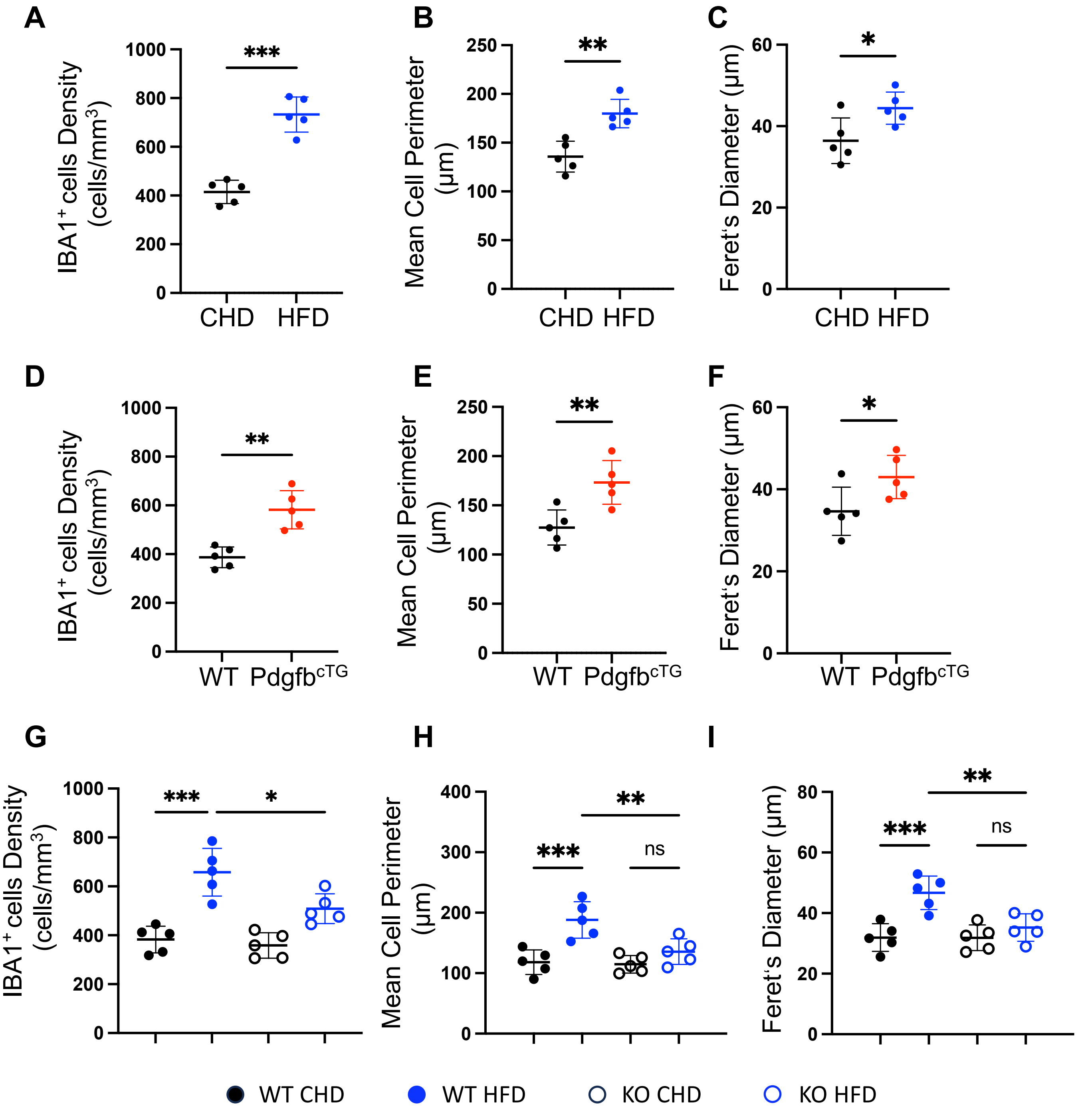

Supplemental Figure 1. Statistic analysis of IBA1+ cells density and microglia morphology. (A-C) Quantification of IBA1+ cells density **(A),** mean cell perimeter (μm) **(B)** and Feret's Diameter (μm) **(C)** in WT CHD and HFD mice. **(D-F)** Quantification of IBA1+ cells density **(D)**, mean cell perimeter (μm) **(E)** and Feret's Diameter (μm) **(F)** in 6-month-old Pdgfb^{cTG} mice and WT littermates. **(G-I)** Quantification of IBA1+ cells density **(G),** mean cell perimeter (μm) **(H)** and Feret's Diameter (μm) **(I)** in Pdgfb^{cKO} mice and WT littermates were fed HFD or CHD for 4 months. n=5. Data are shown as the mean ± SD, *p<0.05, ***p<0.01, ***p<0.001, as determined by unpaired two-tailed Student's *t* test (for two-group comparison) or One-way ANOVA (for multiple-group comparison).
